# The Multidimensional, Intersecting Impacts of COVID-19 on Young People's Lives: Evidence From Cross-Sectional Surveys in Mexico, India, and Kenya

**DOI:** 10.1016/j.jadohealth.2023.06.016

**Published:** 2023-11

**Authors:** Ann Gottert, Isabel Vieitez, René Nevárez, Karen Austrian, Eva Muluve, Sangram K. Patel, Niranjan Saggurti, Ashish Bajracharya, Jessica DeMulder, Erica Soler, Thoai D. Ngo

**Affiliations:** a Population Council, Washington, District of Columbia; b Independent Consultant, Mexico City, Mexico; c Population Council, Mexico City, Mexico; d Population Council, Nairobi, Kenya; e Population Council Institute, New Delhi, India; f Population Council, Dhaka, Bangladesh; g Population Council, New York, New York; h Independent Consultant, Barcelona, Spain

**Keywords:** Intersectionality, Adolescents, Youth, Mental health, Education, Employment, Domestic violence

## Abstract

**Purpose:**

Studies have documented diverse adverse effects of the COVID-19 pandemic on young people's lives—for instance on mental health, education/employment prospects, and intrafamily violence. We sought to generate much-needed evidence regarding whether, and which, young people are experiencing multiple intersecting effects.

**Methods:**

Data come from cross-sectional surveys with young people ages 15–25 years in Mexico (nationwide, n = 55,692), Kenya (four counties, n = 2,750), and India (two states, n = 3,537), collected from late 2020 to early 2022. We used latent class analysis to identify subgroups based on multiple adverse effects, then examined associations between these subgroups and COVID-19 infections/family deaths, and sociodemographic characteristics.

**Results:**

We found prevalent adverse impacts overall and two distinct subgroups in each country—one experiencing higher levels of all impacts, such as on mental health (44%–78% across countries), education/employment (22%–84%), intrafamily violence (22%–49%), and friendships (66%–86%). This subgroup comprised 40% of the sample in Mexico, 25% in Kenya, and 35% in India. In multivariate analyses, this group consistently had greater odds of experiencing COVID-19-related infections and deaths of loved ones. They were more likely socioeconomically disadvantaged, older, urban residents. Associations with other characteristics were country-specific.

**Discussion:**

This study provides novel cross-country evidence that a subgroup of young people has experienced intersecting adverse impacts of COVID-19 on their lives. Findings also confirm prior evidence of multiple elevated vulnerabilities in general. Expanded provision of multiple layers of support is required, particularly for the most vulnerable subgroup, as are multi-sectoral policies and interventions to prevent intersectional effects in future times of crisis.

**Implications and Contribution**

This study is among the first to document the multidimensional, intersecting impacts of the COVID-19 pandemic on young people's lives. Latent class analyses of survey data from Mexico, Kenya, and India identified two distinct subgroups, one experiencing markedly greater impacts on mental health, education/employment, intrafamily violence, friendships, and other dimensions.

There is growing global attention to the adverse impacts of the COVID-19 pandemic on young people's lives, even as this demographic also experiences lower COVID-19-related morbidity and mortality than older generations [1]. Such impacts are likely fueled by mitigation measures such as school closures and service disruptions that have impeded access to critical social, health, and economic resources, as well as by experiences and fears related to COVID-19 infection. Currently, about three years into the pandemic, there is increasingly robust evidence regarding the nature and extent of the diverse impacts of the pandemic among young people [2]. Systematic reviews document consistent study findings of adverse effects on young people's mental health [[3], [4], [5]], educational outcomes [6], and sexual and reproductive health [7]. Studies have also demonstrated other related effects on young people's lives, such as on peer social relationships [8,9] and interpersonal/intrafamily violence [[10], [11], [12]].

Theory and extant research suggest that such vulnerabilities are likely to be intersectional in nature, driven by marginalized identities and socio-structural inequities [[13], [14], [15]], and further amplified by natural disasters and emergencies such as the continuing circumstances of the COVID-19 pandemic [15,16]. Yet to date, there remains a critical gap in the evidence base regarding the extent to which it is the same individuals experiencing intersecting impacts from the COVID-19 pandemic. Most studies focus on one, or only a few, of the areas noted above. And even when studies do focus on multiple such areas, they tend to present prevalence of each outcome separately, as if each operated independently of one another. Taking a simple example, if a study finds that 30% of young people are experiencing adverse impacts on mental health, 30% on violence, and 30% on access to health services, it is often unclear whether it is the same or different individuals experiencing these effects. It also remains unclear whether young people who experience such effects of COVID-19 are also more likely to experience more COVID-19 infection and deaths of loved ones.

In this study, we investigated the intersectional nature of impacts of COVID-19 on the lives of young people in three diverse low-/middle-income countries where our team has worked for decades: Mexico, Kenya, and India. We drew on the five domains of adolescent well-being conceptualized by the United Nations, related to good health (including service access); connectedness and contribution to society; safety and supportive environments; education and employability; and agency and resilience [17]. We hypothesized that the pandemic's impact on such domains are not independent phenomena but rather intersect and demonstrate patterning of who is affected within the population. We also sought to identify characteristics of young people more likely to experience intersecting impacts in each context.

## Methods

In this study, we used latent class analysis (LCA) of cross-sectional survey data to examine the intersectional nature of effects. LCA is a statistically powerful and informative technique that identifies unobserved or hidden profiles within data based on multiple observed variables [18]. LCA can be thought of as a “person-centered” approach to data analysis, in contrast to the dominant “variable-centered” approach that entails describing and assessing associations between individual variables (e.g., regression, structural equation modeling).

Data come from online surveys conducted from November 2020 to February 2021 with 55,692 young people ages 15–24 nationwide across all five regions and 32 states of Mexico; phone surveys conducted in February 2021 with 2,750 young people ages 15–25 in Nairobi, Wajir, Kisumu, and Kilifi counties in Kenya, and phone surveys conducted from December 2021 to January 2022 with 3,537 young people ages 15–25 in Uttar Pradesh and Bihar states in northern India.

Figure A1 shows the severity of the COVID-19 pandemic and stringency of mitigation measures over time in each country using data available online [1], and notes the timing of data collection in each. In general, per capita full-population case prevalence (as well as mortality) has been notably higher in Mexico and India than in Kenya, with multiple waves in each country. Data collection coincided with an oncoming second wave in Mexico and third wave in Kenya, and fell between the largest wave and subsequent second-largest wave in India. The stringency of mitigation measures over time (based on a composite index [19]) was similar in each country, marked by high initial stringency starting in March 2020 (especially in India) and reduced stringency over time (although increased somewhat upon each pandemic wave).

### Participant recruitment and data collection procedures

In Mexico, data come from the first round of *Violence Outcomes in COVID-19 Era Study* (VOCES-19) (Larrea-Schiavon, et al; Unpublished data). Modes of recruitment included social media, newspapers, television/radio, and invitations by educational authorities and youth-serving organizations. Respondents completed the online survey on a personal device.

In Kenya, the sample was drawn from four existing cohorts of young people ages 10–25 years, across both urban informal and rural counties (with over-sampling of females), interviewed by phone in June-August 2020 and February 2021 [11]. Only the latter data, limited to ages 15–25 years, were used for the present analyses, for greater comparability with the other country samples. Respondents without their own phone were reached via their parent/guardian's phone. Seventy percent of the June-August 2020 survey respondents completed the 2021 round.

The sample in India was also drawn from an existing cohort, *Understanding the Lives of Adolescents and Young Adults* (UDAYA) [20,21], a state-level representative longitudinal survey in Uttar Pradesh and Bihar states of young people ages 10–19 years in 2015-16 (with over-sampling of females). Of these households, less than half had phone number on record; of those less than half had functional phone numbers.

More information about methods for each of these studies can be found in published reports [11,[20], [21]] (Larrea-Schiavon, et al; Unpublished data).

### Measures

A detailed description of LCA measures is included in Table A1. Most measures were similar across countries. Validated multi-item measures were employed or adapted to assess concerns related to mental health (depression and/or anxiety) [22,23] and experience of interpersonal violence in the home [24,25], usually complemented by questions asking whether mental health/violence was better/the same/worse for them during the pandemic versus before it. Education/employment, friendships, neighborhood violence, and intimate relationship formation/quality were assessed by questions regarding whether that experience was better/the same/worse compared with prepandemic. We used all binary indicators for the LCA models to facilitate model convergence and interpretation of results [18].

### Data analysis

We conducted all analyses separately by country in Stata v16 (StataCorp LLC, College Station, TX). In Mexico, sampling weights were employed to increase sample representativeness to the population of 15–24-year-olds nationwide, and were constructed based on state of residence, level of rurality of the municipality, sex, and age group.

For the LCA in each country, we started with 6–8 binary indicators. The analysis followed an iterative process entailing constructing a series of models (starting with 1 class and proceeding up to 5, or until models failed to converge), and refining the variables included [18]. Each model yielded probabilities for membership in each class, and, for each class, item response probabilities for each indicator. Final model selection was based on model fit statistics and interpretability (whether the nature of the classes made intuitive sense and were distinct from each other) [18]. We also conducted subgroup analyses by sex/gender identity to gauge differences in the nature of the classes.

Once the final LCA models were selected, we assigned each respondent to one class based on his/her/their highest posterior latent class probability. We then assessed associations between latent class membership and experiences of COVID-19 infection and deaths of close others, as well as sociodemographic characteristics. We conducted both bivariate and multivariate logistic regression analyses, with the latter controlling for sex/gender identity, age (continuous), socio-economic status (SES)/food or household financial insecurity, marital status, and region/county/state.

### Ethical review

A study protocol for each country was approved by the Population Council Institutional Review Board. The study in Kenya was also approved by the Amref Ethical and Scientific Review Committee.

## Results

Table 1 presents sample characteristics for each country (with weighted estimates described henceforth for Mexico). In Mexico, half (50%) of the sample was male, 47% was female, and 3% was nonbinary. About three-quarters (77%) of the sample in Kenya were women, as were 60% in India. Respondents were 19.2 years old on average in Mexico, 18.3 in Kenya, and 21.4 in India. Over 80% in Mexico, 70% in Kenya, and 45% in India were in school when the COVID-19 pandemic began.

**Table 1:** Sample characteristics

	Mexico (n = 55,692)		Kenya (n = 2,750)	India (n = 3,537)
	Weighted % (SE)	Unweighted %	%	%
Sex (Kenya, India)/gender identity (Mexico)				
Male	47.4% (0.55)	37.1%	22.8%	39.8%
Female	50.0% (0.55)	60.1%	77.2%	60.2%
Nonbinary	2.6% (0.17)	2.8%	--	--
Age				
Mean in years (SE/SD)	19.23 (0.03)	16.71 (1.78)	18.3 (2.24)	21.38 (1.98)
15–17 (15–20 in India)	31.4% (0.38)	80.7%	77.7%	33.4%
18–25 (21–25 in India)	68.6% (0.38)	19.3%	22.3%	66.6%
In school when pandemic started	82.6% (0.51)	91.8%	70.2% (5 missing)	45.5%
Married (vs. unmarried)	6.7% (0.37)	1.9%	8.3%	39.2%
Doesn't live with a parent (or in-laws, in India)	33.8% (0.53)	34.6%	21.9%	16.5%
Socioeconomic status or food/household financial insecurity	(tertile)^a^	(tertile)^a^	(Food insecurity)	(Household financial insecurity)
High	36.7% (0.54)	33.3%	47.1%	68.9%
Medium	30.4% (0.50)	33.3%	15.9%	--
Low	32.9% (0.50)	33.3%	37.1%	31.1%
Social media use/smartphone ownership^b^	77.9% (0.46)	80.1%	41.1%	56.6%^a^
Region/County/State				
1	23.3% (0.46)	13.8%	24.3%	53.9%
2	38.8% (0.49)	66.6%	21.1%	46.1%
3	14.5% (0.42)	7.7%	21.0%	--
4	16.7% (0.52)	9.5%	33.7%	--
5	6.7% (0.33)	2.5%	--	--
Urban (vs. rural) area	22.5% (0.46)	10.1%	N/A	34 3%
Indigenous	25.3% (0.47)	23.8%	--	--
Black/Afromexican/Afrodescendent	11.7% (0.34)	11.4%	--	--
Caste	--	--	--	
OBC				58.6%
SC				21.2%
ST				0.6%
General				19.6%
Religion	--	--	--	
Hindu				81.5%
Muslim				18.2%
Other				0.4%

SD = standard deviation; SE = standard error (of weighted estimate).

Regions in Mexico include: (1) South, (2) Center (includes Mexico City), (3) Center-north, (4) North, (5) Northwest.

Counties in Kenya include: (1) Kilifi, (2) Kisumu, (3) Nairobi, (4) Wajir.

States in India include: (1) Uttar Pradesh, (2) Bihar.

a SES tertiles were constructed based on responses to questions adapted from Mexico's National Survey on Income and Expenditures.

b Questions were as follows: uses social media platforms as a way of keeping touch with friends (Mexico); uses at least one of a list of social media platforms regularly (Kenya; n = 1,825, missing for Wajir county); owns his/her/their own smartphone (India).

Less than 10% were married in Mexico and Kenya, while 39% were in India. In each country, over three-quarters currently lived with parents (including in-laws in India). About one-third of the sample in both Kenya and India reported high food/household insecurity. In Mexico, weighted estimate of SES tertiles showed that roughly one-third of the sample fell in each tertile. Regarding social media use, about three-quarters of respondents in Mexico (77%), and 41% in Kenya, reported regularly using social media, and over half (57%) of respondents in India reported owning their own smartphone.

In terms of geographic coverage, the largest proportion of the sample in Mexico (39%) came from the Center region (which includes Mexico City), with at least 7% coming from each of the other four regions in the country. In Kenya and India, residence in each geographic area was roughly evenly split. In each country, one-quarter to one-third were identified as coming from urban (vs. rural) areas. Finally, in Mexico, 25% of respondents identified as indigenous, and 12% as Black, Afromexican, and/or Afrodescendent. And in India, 59% reported belonging to the OBC caste and about 20% the SC caste; 82% reported being Hindu and 18% Muslim.

### Prevalence of adverse effects of COVID-19

Pandemic-related mental health concerns were prevalent (Table 2), particularly in Mexico, where nearly half (48%) of respondents reported such concerns. In Mexico, mean scores on continuous measures (all scaled on a possible range of 0.0–3.0) were 1.03 (standard deviation [SD] 0.81) for depression and 1.06 (SD 0.88) for anxiety. Mean scores were 0.29 (SD 0.48) in Kenya and 0.33 (SD 0.53) in India for a combined depression/anxiety measure. Mental health issues were more prevalent in women, and nonbinary respondents, versus men in Mexico, and were less prevalent among women (vs. men) in India.

**Table 2:** Prevalence of reported adverse impacts of COVID-19 pandemic, overall and by sex/gender identity

	Mexico (n = 55,692)				Kenya (n = 2,750)			India (n = 3,537)		
	Male	Female	Nonbinary	Total	Male	Female	Total	Male	Female	Total
Mental health^a^	40.0%	55.3%∗∗∗	52.0%∗∗	48.1%	18.7%	19.0%	19.0%	36.2%	26.4%∗∗∗	30.3%
Education/employment^b^	16.1%	13.1%∗∗∗	20.0%	14.7%	53.0%	51.9%	52.1%	77.3%	42.0%∗∗∗	56.1%
Intrafamily violence	10.7%	12.0%	15.2%∗	11.5%	16.3%	18.9%	18.3%	13.9%	21.6%∗∗∗	18.5%
Friendships	44.9%	53.9%∗∗∗	48.7%	49.5%	72.4%	67.0%∗	68.3%	35.9%	39.3%∗	38.0%
Access to health services	33.7%	36.2%∗	30.3%	34.9%	11.5%	13.8%	13.3%	--	--	--
Neighborhood violence	15.1%	19.6%∗∗∗	21.5%∗	17.5%	--	--	--	--	--	--
Intimate relationship formation/quality	--	--	--	--	--	--	--	35.5%	21.1%∗∗∗	26.8%

∗*p* < .05 ∗∗*p* < .01 ∗∗∗*p* < .001 for comparison with male, based on bivariate logistic regression analyses.

The analysis in Mexico employed survey weights.

There were no missing data for variables in this table, due in part to the way they were coded (i.e., reporting adverse experience vs. not). The only exception was in Mexico, where the Patient Health Questionnaire-9 (PHQ-9) and General Anxiety Disorder-7 (GAD-7) summary scores (see Table A1) were coded as missing if > 3 of the nine items or >2 of the seven items (respectively) were missing, leading to about 6% of respondents missing on the mental health variable. Stata automatically imputes any missing values using valid responses from other variables. For original variables used to construct LCA variables, missing data were minimal in Kenya (<6%, although violence had up to 8%) and India (all 0%). Missingness ranged from 5% to 13% for each underlying variable in Mexico. This was likely due to the online nature of the survey, thus we believe most missingness was at random.

a Mean scores overall by country on continuous measures (all scaled on a possible range of 0.0–3.0) were 1.03 (SD 0.81) for depression, and 1.06 (SD 0.88) for anxiety in Mexico. Mean scores were 0.29 (SD 0.48) in Kenya and 0.33 (SD 0.53) in India for a combined depression/anxiety measure.

b Only includes education in Mexico, noting 83% were in school at the start of the pandemic.

Adverse pandemic effects on education (and employment, for young adults in India) were reported by over half of respondents in Kenya and India, and 15% in Mexico. Such effects were about 35 percentage points higher among men than women in India, and somewhat more prevalent among men than women in Mexico.

Intrafamily violence was reported by 12% of respondents in Mexico and one-fifth (18%–19%) in Kenya and India. Compared with men, such violence was more prevalent among nonbinary respondents in Mexico and women in India.

Pandemic effects on friendships were reported by over two-thirds (68%) of young people in Kenya, about half in Mexico and over one-third in India. Friendship-related effects were more common among women and nonbinary respondents in Mexico, and women in India (vs. men), and in Kenya were more common among men.

Pandemic effects on access to health services (not assessed in India) were reported by about one-third (35%) of respondents in Mexico (marginally more among women), and 13% in Kenya. Increases in neighborhood violence were reported by 18% of Mexican youth, marginally more by women and nonbinary respondents than men. Finally, pandemic-related intimate relationship formation/quality concerns were reported by 27% of young people in India (the only country where this was assessed at the time)—36% of men and 21% of women.

Missing data were minimal for the above-mentioned variables representing adverse effects of COVID-19 and subsequently empoyed in LCA analyses (missing data are described in detail under Table 2).

### Latent classes characterizing experiences of adverse effects

**Figure 1:**
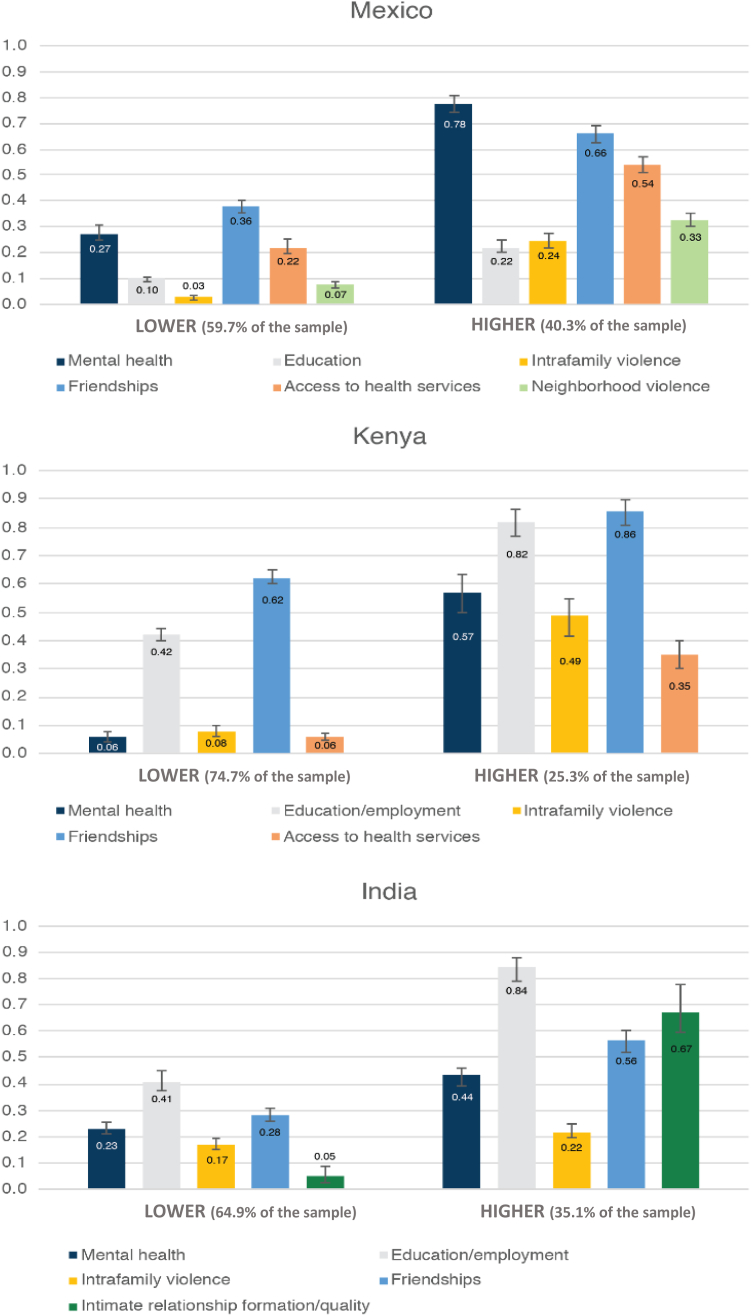
Latent class analysis results, with bars showing item response probabilities.

Two distinct latent classes/subgroups emerged clearly in each country (Figure 1)—one with much higher probabilities of experiencing all adverse impacts than the other. We labeled the subgroups as “Lower” and “Higher.” The Higher subgroup comprised 40% of the sample in Mexico, 25% in Kenya, and 35% in India. Model fit statistics and interpretability together favored the 2-class solution. Akaike Information Criteria and Bayesian Information Criteria statistics (Table A2) showed the steepest drop-off between the 1- and 2-class models and negligible improvements, or slight increases, between the 2- versus 3-class model [26]. Entropy values for 2- and 3-class models were all >0.8 (with values closer to 1.0 representing stronger separation between classes). Four-class models routinely did not converge. For each country, the 3-class models consistently showed a similar pattern as the 2-class models, with all indicators at low/medium/high probabilities, rather than a distinct pattern of item response probabilities (e.g., with certain high and others low, in relation to the other classes). Therefore, we chose the 2-class model in each country for parsimony and ease of interpretation. Additional information about model selection and refinement is available in Table A2.

Indicators included in LCA models across all three countries included mental health, education/employment, intrafamily violence, and friendships; access to health services was also an indicator in Mexico and Kenya. In addition, neighborhood violence was an indicator in Mexico, as was intimate relationship formation/quality in India. All item response probabilities were at least 10% and routinely 20 or 30% higher in the Higher (vs. Lower) subgroup, with many probabilities over 50% in that subgroup.

Before proceeding to the postestimation analyses described in the next sections, we assigned each respondent to one subgroup based on that respondent's highest probability of class membership. Assignment accuracy diagnostics (Table A2) suggested a low probability of misclassification.

### Associations between latent class membership and COVID-19 infections and deaths

In multivariate analyses (Table 3), being in the Higher (vs. Lower) subgroup was associated with nearly 1.5 times the odds of experiencing COVID-19 infections in the household in Mexico (*p* < .001), and twice the odds of knowing others who had COVID-19 in Kenya (*p* < .01). It was also associated with over 1.5 times the odds of experiencing deaths from COVID-19 in the family or of close others in Mexico (*p* < .05) and India (*p* < .01) (not assessed in Kenya).

**Table 3:** Associations between latent class membership and COVID-19 infections and deaths of close others

Values reflect COVID-19 outcomes for the High and Low subgroups	Mexico (n = 55,692)				Kenya (n = 2,750)				India (n = 3,537)			
	Lower	Higher	OR	aOR	Lower	Higher	OR	aOR	Lower	Higher	OR	aOR
Anyone in household diagnosed with COVID-19 (Mexico, India)/Knows anyone who had COVID-19 (Kenya)	18.4% (0.51)	24.8% (0.81)	1.46 ∗∗∗ (1.31, 1.62)	1.47 ∗∗∗ (1.32, 1.65)	2.7%	8.3%	3.22 ∗∗∗ (2.17, 4.78)	2.00 ∗∗ (1.27, 3.15)	2.7%	3.9%	1.47 † (0.99, 2.18)	1.36 (0.91, 2.05)
COVID-19 death(s) of mother, father, sister or brother (Mexico); of close others (India)	0.8% (0.11)	1.2% (0.20)	1.48 † (0.99, 2.21)	1.51^a^ ∗ (1.02, 2.25)	--	--	--	--	4.4%	7.2%	1.69 ∗∗ (1.25, 2.29)	1.64 ∗∗ (1.20, 2.24)

†*p* < .10 ∗*p* < .05 ∗∗*p* < .01 ∗∗∗*p* < .001. OR = odds ratio; aOR = adjusted odds ratio, controlling for sex/gender identity, age, SES/food insecurity/household insecurity, marital status, and region/county/state.

The analysis in Mexico employed survey weights.

a Also controlled for interview date.

### Associations between sociodemographic characteristics and latent class membership

Turning to Table 4, in Mexico, females (adjusted odds ratio [aOR] 1.43, *p* < .001) and nonbinary respondents (aOR 1.53, *p* < .01) had greater odds of being in the Higher subgroup compared with males. In India, compared with males, females had about half the odds of being in the Higher subgroup (aOR 0.56, *p* < .001).

**Table 4:** Associations between latent class membership and sociodemographic characteristics

	Mexico (n = 55,692)			Kenya (n = 2,750)			India (n = 3,537)		
	% In Higher subgroup (vs. Lower)	OR	aOR	% In Higher subgroup (vs. Lower)	OR	aOR	% In Higher subgroup (vs. Lower)	OR	aOR
Gender identity (Mexico)/Sex (Kenya, India)									
Male	30.3%	ref	ref	21.1%	ref	ref	40.8%	ref	ref
Female	38.9%	1.46 ∗∗∗ (1.32, 1.61)	1.43 ∗∗∗ (1.30, 1.58)	24.0%	1.18 (0.95, 1.47)	0.88 (0.68, 1.15)	22.9%	0.43 ∗∗∗ (0.37, 0.50)	0.51 ∗∗∗ (0.43, 0.60)
Nonbinary	37.6%	1.38 ∗ (1.04, 1.84)	1.53 ∗∗ (1.13, 2.05)	--			--		
Age in years									
15-17 (15-19 in India)	24.6%	ref	Ref	18.1%	ref	Ref	32.3%	ref	ref
18-25 (21-25 in India)	39.4%	1.99 ∗∗∗ (1.85, 2.14)	1.98 ∗∗∗ (1.84, 2.13)	41.4%	3.19 ∗∗∗ (2.62, 3.87)	1.80 ∗∗∗ (1.37, 2.28)	28.8%	0.85 ∗ (0.73, 0.99)	0.97 (0.83, 1.15)
Region/County/State									
1	34.7%	ref	ref	22.2%	ref	Ref	29.2%	ref	ref
2	37.7%	1.14∗ (1.02, 1.27)	1.17∗∗ (1.05, 1.31)	45.6%	2.94 ∗∗∗ (2.30, 3.76)	1.76 ∗∗∗ (1.33, 2.32)	33.0%	1.19 ∗ (1.03, 1.38)	1.22 ∗ (1.05, 1.42)
3	34.7%	1.00 (0.85, 1.18)	1.03 (0.87, 1.22)	34.0%	1.81 ∗∗∗ (1.41, 2.33)	2.28 ∗∗∗ (1.72, 3.01)			
4	29.2%	0.78 ∗∗ (0.64, 0.93)	0.82∗ (0.68, 0.99)	3.5%	0.13 ∗∗∗ (0.08, 0.19)	0.16 ∗∗∗ (0.11, 0.24)			
5	32.6%	0.91 (0.72,1.15)	0.96 (0.75, 1.22)						
Urban area (vs. rural)									
No	31.4%	1.22 ∗∗∗ (1.09, 1.37)	1.28 ∗∗∗ (1.13, 1.44)	N/A	N/A	N/A	29.0%	1.30 ∗∗ (1.12, 1.50)	1.04 (0.88, 1.23)
Yes	35.8%						34.7%		
Married (vs. unmarried)	(insufficient sample size)								
No				22.1%	2.01 ∗∗∗ (1.51, 2.68)	1.24 (0.84, 1.82)	36.1%	0.53 ∗∗∗ (0.46, 0.62)	0.73 ∗∗ (0.60, 0.88)
Yes				36.4%			23.1%		
Doesn't live with a parent									
No	33.7%	1.16 ∗∗ (1.05, 1.28)	1.06 (0.95, 1.17)	20.5%	1.93 ∗∗∗ (1.58, 2.35)	0.98 (0.74, 1.29)	30.5%	0.80 ∗ (0.65, 0.98)	0.77 ∗ (0.63, 0.95)
Yes	37.0%			33.2%			26.0%		
In school when pandemic started									
No	36.8%	0.94 (0.81, 1.08)	1.43 ∗∗∗ (0.22, 1.68)	33.5%	0.47 ∗∗∗ (0.39, 0.56)	0.65 ∗∗ (0.50, 0.84)	22.7%	2.23 ∗∗∗ (1.92, 2.59)	2.20 ∗∗∗ (1.85, 2.61)
Yes	35.2%			19.1%			39.7%		
Socioeconomic status									
High	32.7%	ref	ref	11.7%	ref	ref	30.8%	ref	ref
Medium	33.7%	1.05 (0.93,1.17)	1.05 (0.93,1.18)	29.4%	3.13 ∗∗∗ (2.38, 4.12)	2.62 ∗∗∗ (1.93, 3.55)	--	1.03 (0.86, 1.23)	1.29 ∗∗ (1.07, 1.56)
Low	38.0%	1.26 ∗∗∗ (1.12, 1.41)	1.22 ∗∗∗ (1.09, 1.37)	36.6%	4.34 ∗∗∗ (3.49, 5.40)	3.08 ∗∗∗ (2.43, 3.92)	3. 5%		
Social media user				(n = 1,823)	(n = 1,823)	(n = 1,823)			
No	38.0%	0.87 ∗ (0.78, 0.98)	0.85 ∗∗ (0.76, 0.96)	30.4%	1.39 ∗∗ (1.14, 1.70)	0.91 (0.71, 1.17)	22.8%	2.01 ∗∗∗ (1.73, 1.33)	1.38 ∗∗ (1.15, 1.65)
Yes	34.8%			37.8%			37.2%		
Indigenous		1.06 (0.96, 1.18)	1.04 (0.93, 1.15)	N/A	N/A	N/A	N/A	N/A	N/A
No	34.8%								
Yes	36.2%								
Black/Afromexican/Afrodescendent		1.07 (0.94, 1.23)	1.14 (0.99, 1.32)	N/A	N/A	N/A	N/A	N/A	N/A
No	35.0%								
Yes	36.6%								
Caste	N/A	N/A	N/A	N/A	N/A	N/A			
OBC							30.4%	(ref)	(ref)
SC							30.5%	1.01 (0.84, 1.21)	1.00 (0.82, 1.21)
ST							40.0%	1.53 (0.62, 3.75)	1.39 (0.52, 3.56)
General							32.9%	1.12 (0.93, 1.35)	1.10 (0.91, 1.34)
Religion	N/A	N/A	N/A	N/A	N/A	N/A			
Hindu							31.8%	(ref)	(ref)
Muslim							27.7%	0.82 ∗ (0.68, 0 99)	0.88 (0.71, 1.08)
Other							14.3%	0.36 (0.08, 1.60)	0.41 (0.09, 1.83)

∗*p* < .05 ∗∗*p* < .01 ∗∗∗*p* < .001. OR = odds ratio; aOR = adjusted odds ratio, controlling for sex/gender identity, age (continuous), SES/food insecurity/household insecurity, marital status, and region/county/state.

Regions in Mexico include: (1) South, (2) Center (includes Mexico City), (3) Center-north, (4) North, (5) Northwest.

Counties in Kenya include: (1) Kilifi, (2) Kisumu, (3) Nairobi, (4) Wajir.

States in India include: (1) Uttar Pradesh, (2) Bihar.

The analysis in Mexico employed survey weights.

Older respondents (ages 18–24/25, vs. 15–17 years) in Mexico and Kenya had nearly twice the odds of being in the Higher subgroup (aORs 1.98 and 1.80, respectively, both *p* < .001); no age difference was evident in India (noting that the age distribution was higher than the other two countries). The effects of marital status, living with parent(s), and being in school when the pandemic began varied by country. In Mexico and India, being in school was associated with 1.4 and 2.2 greater odds, respectively, of being in the Higher subgroup (both *p* < .001), whereas in Kenya, there was a protective effect (aOR 0.65, *p* < .01). And in India, there was about a 25% protective effect of being married (*p* < .01) and not living with parent(s)/in-laws (*p* < .05).

Lower SES/higher food/household financial insecurity was associated with greater odds of Higher subgroup membership in each country (*p* < .01–<.001). Regarding social media use/smartphone ownership, there was a protective effect in Mexico (aOR 0.85, *p* < .01) and a harmful effect in India (aOR 1.4, *p* < .01) (no effect in Kenya).

There were also notable geographic differences in class membership within each country. In Mexico, the Center region (home of Mexico City) had the greatest odds of respondents being in the Higher subgroup, as did living in an urban area (regardless of region, *p* < .001). In Kenya, compared with Kilifi county (largely rural), the more urban Nairobi and Kisumu counties had elevated odds of residents belonging to the Higher subgroup (aOR 2.3 and 1.8, respectively, both *p* < .001), whereas Wajir (rural) had much lower odds (0.16, *p* < .001) (residence in urban vs. rural *subareas* of each county was not assessed in Kenya.). And in India, respondents from Bihar (the more urban of the two states) had 22% greater odds of Higher subgroup membership than Uttar Pradesh (*p* < .05), with urban residence trending in that direction as well.

## Discussion

Findings from this study align with those from other global research documenting diverse elevated vulnerabilities among young people during the COVID-19 pandemic. This study also provides clear, novel cross-country evidence that a subgroup of young people has experienced intersecting adverse impacts of COVID-19 on their lives. In each country, one of the two identified subgroups experienced markedly higher prevalence across all adverse effects—including mental health, education/employment, intrafamily violence, and friendships. Still, it is important to note that the other subgroup had non-negligible levels of adverse impacts in each country; that is, it is not that members of the other subgroup were not impacted, just much less so than the other subgroup. Young people experiencing these co-occurring effects were also more likely to have experienced COVID-19 infection and deaths of loved ones. Experiencing such intersecting vulnerabilities is fundamentally different, and greater, than the sum of its parts, and thus any response that does not take such intersectionality into account will be insufficient [13,15,16].

Regarding which young people are experiencing intersecting effects, we found that certain characteristics—namely socioeconomic disadvantage, older age, and urban residence—demonstrated consistent adverse negative across countries, while others differed substantially by country. Socioeconomic disadvantage and inequality is perhaps the most well-documented driver of intersectional vulnerabilities [14], including in the context of COVID-19 [27,28]. Older age has emerged in several COVID-19 era studies among youth as correlating with a range of adverse effects, particularly on mental health [[4], [5], [6]]. The reasons for this likely differ by outcome and merit further investigation. Young people experience multiple, often rapid transitions such as leaving school and/or their household, and entering employment and intimate relationships, perhaps leading to new vulnerabilities as well as new (sometimes unmet) expectations due to COVID-related restrictions. The consistent associations between urban residence and greater vulnerability also require further exploration. Finally, the geographic variation of adverse COVID-19 impacts may suggest social or structural drivers at play.

The differing associations between sex/gender identity and experiences of intersecting vulnerability by country were somewhat surprising. In Mexico, both women and nonbinary respondents were more likely than men to be in the Higher subgroup, in line with most extant research showing women and girls' magnified vulnerabilities during the pandemic [5,11,29], as well as research around experiences of sexual and gender minorities [30,31]. Conversely, the finding that, in India, men were more likely than women to be in the Higher subgroup may reflect harsh economic impacts of early COVID-19 mitigation measures in that country [32] (which likely disproportionally affects men given their much higher participation in the workforce than women [33]), and associated impacts on mental health and ability to from intimate relationships.

Other characteristics that diverged between countries in their associations with membership in the Higher subgroup included being in school at pandemic start, marital status, living with parents, and social media use. The harmful effect of being in school in Mexico and India could reflect that group's experience of schooling disruption as well as thwarted educational hopes, with concomitant effects on mental health and friendships. In Kenya, at the time of data collection (early 2021), students were back in school (many at boarding schools, common in that country) after long closures, potentially allowing them to access amenities and protections often not available at home. Finally, the contrasting effects of social media use between countries also require further investigation.

Targeted policies and social protection programs are needed to provide multiple layers of support, particularly to young people experiencing multiple intersecting vulnerabilities, building on but extending well beyond existing social safety nets. [[34], [35], [36]] In each country, there emerged remarkably high prevalence of specific adverse pandemic impacts (in the Higher subgroup, and at times overall), warranting immediate response. We would highlight impacts on mental health in Mexico, education/employment in India and Kenya, and intrafamily violence in Kenya—as detailed in our team's other publications [10,11,20,37,38]. Responses could include strengthening and expanding phone and online helplines and services, community-based services, and support groups, and ensuring these resources are youth-friendly and have appropriate referrals between them.

Alongside targeted social protection and support provision, more forward-looking policy change is also required to prevent such impacts from occurring in the future. The intersectional nature of adverse impacts of COVID-19, and structural and social determinants that drive them, should guide decision-making about the design and resourcing of inclusive social protection measures, public health interventions, and public policies, including those to promote the well-being of the largest-ever generation of adolescents and young people [5].

This study has several limitations. First, we urge caution in directly comparing findings between the three countries given differences in timing of data collection (and hence epidemic stage and nature of the response), recruitment strategy, and certain measures. Second, the cross-sectional nature of the data (and some degree of ambiguity regarding when during the pandemic reported effects occurred) limits inferences about causality. Third, heterogeneity between countries in how each variable was measured could have influenced latent class findings. Fourth, all data are based on self-report, which could be subject to recall and/or social desirability bias. Fifth, given the mode of data collection, youth without internet/phone access may have been less-reached, potentially underrepresenting a more vulnerable population. Finally, findings may not be generalizable to the full populations of young people ages 15–24/25 years in each country (or pertinent region/state/county), nor beyond the three countries.

In conclusion, this study provides the type of robust evidence sorely needed if intersectionality is to be taken seriously in social protection and structural change responses. We encourage investigations of intersectional impacts of COVID-19, through new research initiatives as well as analyses of existing data. Such research could help propel the kind of primary prevention and structural change efforts just described and ensure that such efforts to build back are inclusive of young peoples' diverse voices. Investing in studying the pandemic's longer-term impacts and testing solutions to support recovery will likewise be critical.

## Supplementary Data

Figure A1 and Table A1-A2
