# Supplementary material for: The Multidimensional, Intersecting Impacts of COVID-19 on Young People's Lives: Evidence From Cross-Sectional Surveys in Mexico, India, and Kenya
Source: J Adolesc Health. 2023 Nov;73(5):820–9. doi: 10.1016/j.jadohealth.2023.06.016 (PMC10581329; doi:10.1016/j.jadohealth.2023.06.016)
Supplement: Figure A1 and Table A1-A2 [file mmc1.docx]

**
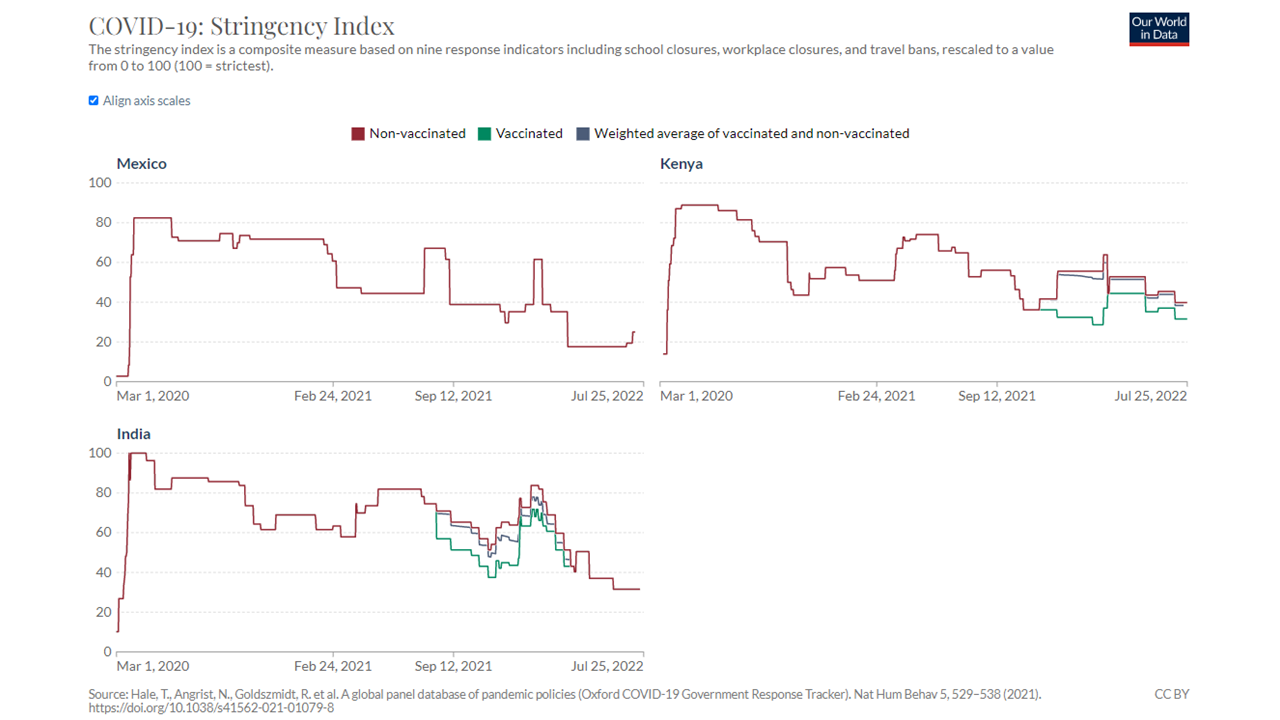

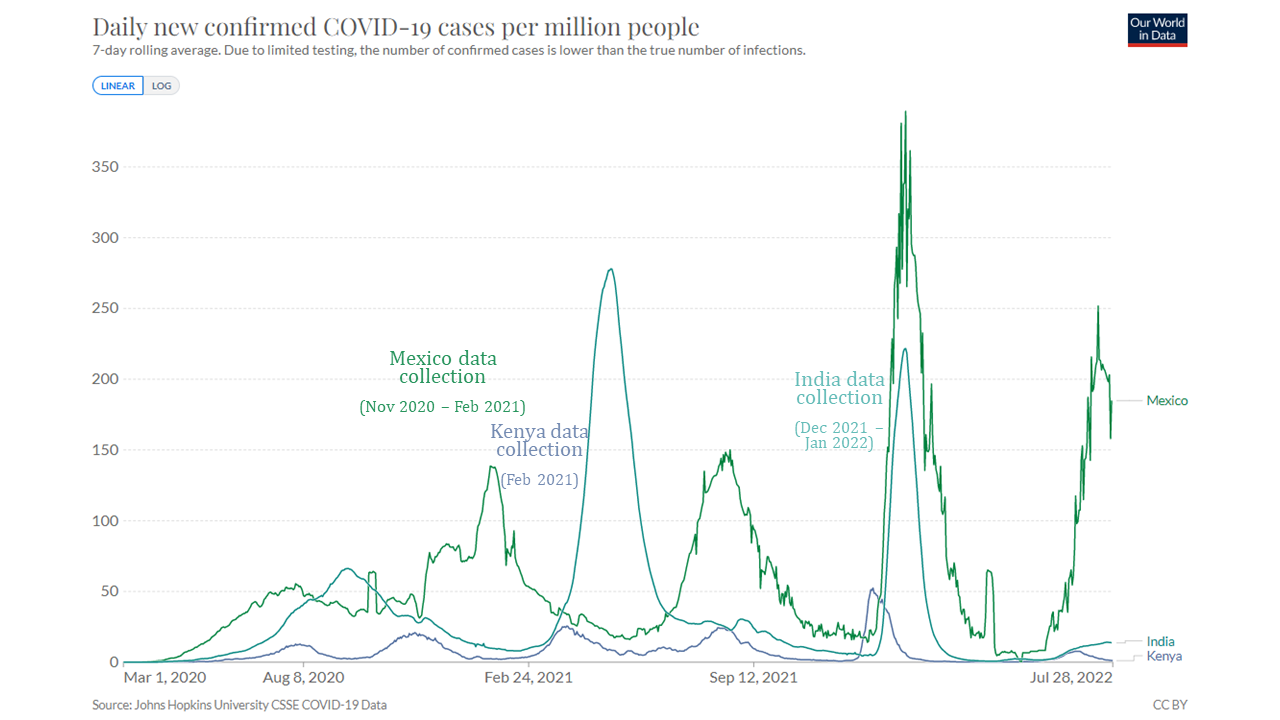
**

Ritchie, H., Mathieu, E., Rodés-Guirao, L., et al. Coronavirus Pandemic (COVID-19). OurWorldInData.org. 2020. Available from: https://ourworldindata.org/coronavirus.

**Figure S1. Severity of COVID-19 pandemic and stringency measures over time**

**Table S1. Measures**

|  | **Mexico** | **Kenya** | **India** |
| --- | --- | --- | --- |
| **Mental health** | Defined as at least mild depression per PHQ-9 scale^1^, OR at least mild anxiety per GAD-7 scale^2^, AND, after these scales, when asked “Thinking about before COVID, have these feelings been bothering you more, the same, or less?” answered “A bit more than before” or “Much more than before”. | Binary; defined as at least mild depression/anxiety per PHQ-4 scale^3^ | Binary; defined as at least mild depression/anxiety per PHQ-4 scale^3^ and AND, after this scale, when asked “Thinking about before COVID, have these feelings been bothering you more, the same, or less?” answered “A bit more than before” or “Much more than before”; OR responded “some of the time”, “often”, or “very often” to “During the COVID-19 pandemic, in general how often have you felt isolated or lonely?” |
| **Education/**  **employment** | Responded "Yes, less school likely” to the question “Since the school closure, have your schooling plans changed?" | Among those who were in school before COVID, reported not currently being enrolled in school or enrolled but not attending and not learning from home OR responded "very true" to the question “Most classmates that I was in school with before Corona have not returned” (suggesting context of school-leaving), OR among those who reported doing any activities to earn money in the last month, reported earning less than before Corona, looking for work but not being successful (in last 6 months), or having less control over earning money | When asked “Please answer whether your ability to meet the following needs was negatively affected, not affected, or positively affected by the COVID-19 pandemic, in the last year”, responded “Negatively affected” to “Your desire to complete more education (now and in the future)” or “Your hopes for work/profession (now and in the future)” |
| **Intrafamily violence** | Among those who reported experiencing emotional violence (“humiliated, insulted, or threatened”), physical violence (“someone in your household pushed, slapped or done other physical aggression to you”), or sexual violence (“forced to do something sexually that you would not have wanted to do”) in the last month, responded “More” to the question “Would you say that it is happening more, less or the same as compared to before Corona started?” Measure adapted from the COVID-19 Impact on Health and Wellbeing Survey (University of Texas).^4^ | Among those who reported experiencing emotional violence (“humiliated, insulted, or threatened”), physical violence (“someone in your household pushed, slapped or done other physical aggression to you”), or sexual violence (“forced to do something sexually that you would not have wanted to do”) in the last month, responded “More” to the question “Would you say that it is happening more, less or the same as compared to before Corona started?’” OR reported that there is “More” “Tensions in my house”, “My parents/guardians or other adults in the household arguing” or “People being beaten in my home”, as one of a series of statements about different aspects of life that may have been affected by Corona. Measures adapted from two sources.^4,5^ | Responded “Yes” to “Since COVID began, has someone in your household insulted, yelled at, or humiliated you?” or “Since COVID began, has someone in your household pushed, slapped, or otherwise been physically aggressive with you?” or “Would you say there is more violence in your household during COVID-19, compared with before?” Measures adapted from two sources.^4,5^ |
| **Friendships** | Reported that “the amount of time I spend with friends” was less than before COVID” was “less than before” (vs. “the same” or “more”) AND reported having <=3 "friends you have that you could call on for help" | Reported that “the amount of time I spend with friends” was less than before COVID (vs. “the same” or “more”) | Reported that “you are keeping contact with your friends” was less than before COVID (vs. “the same” or “more”) |
| **Access to health services** | Responded 'Yes, a lot' or 'Yes, to some extent' to the question “Since the start of the pandemic, do you think your access to health services (family medicine, already scheduled appointments, etc) has been affected?” | Answered “Yes” to “Was there a time in the past month that you were sick, needed a health service, medicine or product and you did not get it?” | N/A |
| **Neighborhood violence** | Reporting that there is “More” “Crime or violence in my neighborhood”, as one of a series of statements about different aspects of life that may have been affected by Corona OR responding "Not very safe' or 'Not safe at all' to the question "Since COVID, how safe do you feel in your neighborhood?" | N/A | N/A |
| **Intimate**  **relationship**  **formation/quality** | N/A | N/A | When asked “Please answer whether your ability to meet the following needs was negatively affected, not affected, or positively affected by the COVID-19 pandemic, in the last year”, responded “Negatively affected” to “Your relationship with intimate partner(s)” or “Your ability to find an intimate partner/meet new partners” |

1. Kroenke K, Spitzer RL, Williams JB. The PHQ‐9: validity of a brief depression severity measure. Journal of general internal medicine. 2001;16(9):606-13.

2. Spitzer RL, Kroenke K, Williams JB, Löwe B. A brief measure for assessing generalized anxiety disorder: the GAD-7. Archives of internal medicine. 2006;166(10):1092-7.

3. Kroenke K, Spitzer RL, Williams JB, Löwe B. An ultra-brief screening scale for anxiety and depression: the PHQ–4. Psychosomatics. 2009;50(6):613-21.

4. Jetelina KK, Knell G, Molsberry RJ. Changes in intimate partner violence during the early stages of the COVID-19 pandemic in the USA. Injury prevention. 2021;27(1):93-7.

5. Grasso DJ, Briggs-Gowan MJ, Ford JD, Carter A. The epidemic–pandemic impacts inventory (EPII). University of Connecticut School of Medicine. 2020.

**Supplemental Table S2. Model fit statistics and class assignment diagnostics**

|  | **Mexico**  (n=55,692) | |  | **Kenya**  (n=2,750) | |  | **India**  (n=3,537) | |  |
| --- | --- | --- | --- | --- | --- | --- | --- | --- | --- |
| **Goodness-of-fit statistics** | AIC | BIC | Entropy | AIC | BIC | Entropy | AIC | BIC | Entropy |
| 1-Class | 360783.03 | 360836.60 | N/A | 14697.65 | 14727.25 | N/A | 21595.27 | 21626.12 | N/A |
| 2-Class | 352520.59 | 352636.70 | 0.848 | 14147.66 | 14212.77 | 0.888 | 21040.29 | 21114.34 | 0.880 |
| 3-Class | 352178.49 | 352357.00 | 0.842 | 14146.10 | 14246.73 | 0.825 | 20984.81 | 21102.06 | 0.893 |
|  | | | | | | | | | |
| **Assignment accuracy diagnostics**  Final classes: | Proportion assigned to class | AvePP |  | Proportion assigned to class | AvePP |  | Proportion assigned to class | AvePP |  |
| *Low* | 0.7213^a^ | 0.816 |  | 0.7669 | 0.915 |  | 0.6904 | 0.873 |  |
| *High* | 0.2787^a^ | 0.804 |  | 0.2331 | 0.805 |  | 0.3096 | 0.851 |  |

AIC=Akaike Information Criteria, BIC=Bayesian Information Criteria, with lower values signifying a better fit.

Regarding Entropy, the closer the value is to 1, the stronger the separation between classes.

AvePP=Average Posterior Probability of Assignment, ≥0.70 indicates high assignment accuracy.

^a^ Proportion assigned to class in Mexico is substantially different from the probability of class membership, likely due to inability to weight the

assignment of respondents to class; weights are applied in postestimation analyses.

Regarding selection/refinement of final indicators for LCA models, most that were entered into initial models consistently aligned with the low/high pattern. Exceptions were household food/financial insecurity during COVID-19, and access to sexual and reproductive health services, which in each country did not consistently conform to this pattern nor exhibit a clear pattern in relation to the other indicators and was thus removed from the models. Regarding differentiation of latent class models by sex/gender identity, in each country we found a similar pattern by which, while there was some degree of differences in magnitude of item response probabilities, the nature of the subgroups – one lower across the board and one substantially higher – remained. Therefore, we chose to examine sex/gender identity differences in post-estimation analyses rather than estimating such differences as part of the LCA model itself.

.
